# THINGSplus: New norms and metadata for the THINGS database of 1854 object concepts and 26,107 natural object images

**DOI:** 10.3758/s13428-023-02110-8

**Published:** 2023-04-24

**Authors:** Laura M. Stoinski, Jonas Perkuhn, Martin N. Hebart

**Affiliations:** 1 Max Planck Institute for Human Cognitive & Brain Sciences, Leipzig, Germany; 2 Justus Liebig University, Gießen, Germany

**Keywords:** Database, Object concepts, Object images, Concrete concepts, Semantic norms, Visual norms, Object features

## Abstract

To study visual and semantic object representations, the need for well-curated object concepts and images has grown significantly over the past years. To address this, we have previously developed THINGS, a large-scale database of 1854 systematically sampled object concepts with 26,107 high-quality naturalistic images of these concepts. With THINGSplus, we significantly extend THINGS by adding concept- and image-specific norms and metadata for all 1854 concepts and one copyright-free image example per concept. Concept-specific norms were collected for the properties of real-world size, manmadeness, preciousness, liveliness, heaviness, naturalness, ability to move or be moved, graspability, holdability, pleasantness, and arousal. Further, we provide 53 superordinate categories as well as typicality ratings for all their members. Image-specific metadata includes a nameability measure, based on human-generated labels of the objects depicted in the 26,107 images. Finally, we identified one new public domain image per concept. Property (M = 0.97, SD = 0.03) and typicality ratings (M = 0.97, SD = 0.01) demonstrate excellent consistency, with the subsequently collected arousal ratings as the only exception (*r* = 0.69). Our property (M = 0.85, SD = 0.11) and typicality (*r* = 0.72, 0.74, 0.88) data correlated strongly with external norms, again with the lowest validity for arousal (M = 0.41, SD = 0.08). To summarize, THINGSplus provides a large-scale, externally validated extension to existing object norms and an important extension to THINGS, allowing detailed selection of stimuli and control variables for a wide range of research interested in visual object processing, language, and semantic memory.

## Introduction

There are a large number of different objects in the world, and researchers from various disciplines have made great efforts to understand how they are processed and semantically represented in memory (DiCarlo et al., 2012; Grill-Spector & Weiner, 2014; Lambon-Ralph, 2014). Given the endless scope of possible object categories, selecting a well-curated set of object concepts is crucial for systematically investigating object recognition or semantic memory.

Many researchers manually curate stimuli for their experiments, which requires a massive investment of time and effort and risks creating a limited and unrepresentative selection of concepts. Others make use of existing databases comprising pre-curated sets of concepts or images. However, many of these databases have shortcomings that make them insufficiently suited for research in vision science. For instance, they may be based on a comparably small number of objects cropped from their natural background (e.g., *BOSS*: Brodeur et al., 2014), contain objects that have been selected in a more-or-less arbitrary fashion for the purpose of image classification, and contain images of insufficient quality for psychological and neuroscience experiments (e.g., *ImageNet*: Deng et al., 2009; for a review, see Hebart et al., 2019).

To provide an alternative to classical object concept and image databases, we developed the *THINGS database* (Hebart et al., 2019; https://osf.io/jum2f/). THINGS is a comprehensive, freely available database of 1854 living- and non-living objects systematically sampled from American English, with a minimum of 12 images per object concept, consisting in total of 26,107 naturalistic, high-quality object images. In addition, THINGS includes validated category memberships of the concepts for the 27 most common higher-level categories (e.g., “animal,” “food,” “furniture”).

Most importantly, THINGS distinguishes itself in its extensive and systematic selection of concrete object concepts. Sampling concepts from almost all nameable objects in the world is crucial to ensure a comprehensive representation of the entire object space. THINGS was created by collecting a list of concrete, picturable object nouns from an existing word database (Brysbaert et al., 2014). Synonymous words were unified, and we applied crowdsourcing to further reduce our selection to concepts nouns that were named consistently. This approach ensured a sufficiently comprehensive selection while avoiding redundancy and concepts too specific to be robustly identified. For example, we excluded the concept “robin” since it was consistently named “bird”.

The THINGS image dataset comprises natural and colored photographs of object concepts, which pose a more naturalistic depiction of everyday objects than line drawings or images with the objects isolated from their background (Bracci et al., 2017; Bracci & de Beeck, 2016; Proklova et al., 2016). We also focused on images showing one prominent, picturable object contrary to images displaying multiple concepts (e.g., as in a still-life) and to natural, navigable scenes (e.g., cities, beaches). We concentrated on concrete and individually depicted concepts, as they are the main subject of much research regarding object processing and semantic memory. Further, our dataset offers several images per concept, necessary to determine robust and generalizable object representations. Lastly, we curated the images manually and defined several selection criteria to assure a standardized and high quality of our images (see Hebart et al., 2019, for details).

Together, THINGS provides a rich resource of systematically selected object concepts, images, and high-level categories. As such, THINGS offers a valuable tool optimized for systematic and large-scale naturalistic research in psychology, neuroscience, and computer science. It supports researchers in selecting a representative and standardized set of object concepts and images, providing a foundation for exploring the perceptual and cognitive processing of complex real-world stimuli at scale or with a systematic sampling strategy. In addition, with THINGS starting to be adopted more widely (e.g., de Varda & Strapparava, 2022; Demircan et al., 2022; Dobs et al., 2022; Frey et al., 2021; Gifford et al., 2022; Griffin, 2019; Grootswagers et al., 2022; Lam et al., 2021; Muttenthaler et al., 2022; Ratan Murty et al., 2021; Rideaux et al., 2022), THINGS allows increased comparability between studies across different laboratories or disciplines.

### The need for extending the THINGS database

Yet, more work is required to further develop and improve the THINGS database. For instance, while THINGS was published according to fair use in the United States and as such may be used for research purposes (https://www.copyright.gov/fair-use/), any copyright restrictions, including the *creative commons licenses* (https://creativecommons.org/) will also impose restrictions on the use of image databases in publications. Thus, while mostly unlimited use of THINGS is possible for research purposes, there are still restrictions for visualizing THINGS images in publications. Thus, providing a set of public domain images would allow the free usage and editing of those images while increasing the scope of the THINGS image dataset, specifically for data driven analyses that in part depend on being able to visualize images.

THINGS includes membership information for 27 common higher-level categories that merely encompass around half of the object concepts. Identifying additional categories would allow for finer distinctions between objects and facilitate selecting or excluding stimuli representing specific object classes (e.g., choosing only living or non-living objects). Further, it would open up a way to investigate category-specific effects for a larger number of objects and object domains.

Typicality ratings are often used in psychological research to judge the degree of representativeness of an object for higher-level categories. While some objects are considered good or typical members of a category, others are perceived to be less typical members (e.g., an apple is a more typical fruit than a coconut; Rosch, 1975). Typicality is of major interest when selecting object concepts, as typical examples of a category are preferentially processed over atypical members (Larochelle & Pineau, 1994; Rosch & Mervis, 1975; Woollams, 2012). Gathering typicality ratings on THINGS concepts would thus enormously improve their applicability for research on object categorization and semantic knowledge more generally.

Also, objects can be characterized according to many possible criteria. For example, neuropsychological and neuroimaging evidence suggest that dimensions such as animacy (e.g., *is it alive?*), real-world size (e.g., *what size does this object usually have in real life*?), and manipulability (e.g., *how easily can you grasp it?*) play a critical role in mental object representation (Caramazza & Shelton, 1998; Chao et al., 1999; Sudre et al., 2012). Other critical dimensions include movability (e.g., *can it move?*) and naturalness (e.g., *is it manmade or natural?*; Huth et al., 2012; Magri et al., 2020; Sudre et al., 2012). Further, object images and concepts vary in their subjective value, weight, emotional valence, or arousal (Bradley & Lang, 1999; Sudre et al., 2012). Object ratings along these dimensions would enhance their interpretability and allow for identifying properties underlying mental and neural representations of objects. These and similar properties have been collected for other smaller-scale image databases (e.g., Brodeur et al., 2014) but not for THINGS concepts or images.

Finally, labels for the object concepts in THINGS were generated based on an existing word database - *WordNet* (Fellbaum, 1998). The nameability of the 1854 concept nouns was verified by asking humans to label the objects based on their natural appearance in individual photographs. It is, however, unclear to what degree the desired concepts are correctly named in all 26,107 images of the database. For example, many concepts have synonymous designations, and while the concept “couch” might be named correctly in some of the THINGS images, it might be consistently called “sofa” in others. Providing a measure of concept nameability would quantify the extent to which the THINGS nouns pose the appropriate label for each of their corresponding images. In addition, it would be helpful to determine how people generally call the concepts and how strongly they agree on this.

### Aim of the THINGSplus project

The general aim of *THINGSplus* is to extend and improve the THINGS database to increase its utility for research communities in psychology, neuroscience, and computer science. We intend to provide researchers in those fields with concept-specific norms and metadata, including (1) an expanded set of 53 higher-level categories, (2) typicality ratings of object concepts within these categories, and (3) evaluations of concepts along critical object dimensions (e.g., size, animacy, manmadeness). Further, we extended the THINGS image dataset by (4) collecting human-generated labels of objects based on their appearance in naturalistic images and (5) providing one additional public domain image per concept.

To create norms and metadata, we conducted four short experiments on the online crowdsourcing platform Amazon Mechanical Turk (AMT). In Experiment 1, we asked AMT workers to label the main object and other potential objects in all 26,107 images using one word. In Experiment 2, participants sorted objects based on their typicality for 53 higher-level categories. Finally, in Experiment 3, we collected ratings of real-world size and size range of object concepts and in Experiment 4 asked participants to classify the degree to which objects relate to 11 critical object dimensions “manmadeness," "preciousness,” “liveliness,” “heaviness,” “naturalness,” “ability to move,” “graspability,” “holdability,” “ability to be moved,” “pleasantness,” and “arousal level”. Public domain images and membership affiliation to newly identified higher-level categories were manually selected by the authors.

All norms, metadata, and supplementary public domain images of the THINGSplus project have been added to the existing THINGS database, which is freely available for academic purposes (https://osf.io/jum2f/).

THINGSplus provides a substantial extension of THINGS, but also offers unique value beyond other existing databases. Most importantly, the number of object concepts and images captured by our norms are much broader than those found in existing databases and include a systematically selected set of object categories not typically covered in other datasets. Thus, it is much more likely for authors to find norms for their object categories and facilitate the selection of a standardized set of concepts, images, and control variables according to a researcher’s individual needs. In addition, our comprehensive database allows a much broader study of the effects of individual object-related variables than previously possible and allows assessing how previous findings may generalize to a larger set of objects.

## Methods

### Selection of public domain images

The THINGS image dataset includes a comprehensive set of naturalistic photographs, with 12 or more example images per concept. However, since most images in THINGS are not from the public domain, it is challenging to use them as example images in publications. To overcome this issue, we identified one additional public domain image for each of the 1854 object concepts. Identification of candidate images and postprocessing (e.g., cropping) were carried out by the authors in multiple steps (see below). This selection process was repeated until one suitable image was identified per concept.

#### Selection criteria

We selected one novel, freely usable (i.e., public domain) picture for every object concept. The images were colored photographs of one or multiple examples of the respective object cropped to a square size. The selection criteria followed identical guidelines as reported in Hebart et al. (2019). For some concepts, it was challenging to find appropriate images that conformed to our selection criteria. If our web search resulted in no suitable candidate image, we either loosened the criteria slightly or took our own pictures (see below for details). To make these decisions transparent, we distinguish between the terms “exclusion” and “avoiding.” The former refers to strictly observed exclusion criteria, whereas the latter describes guidelines that were less strictly adhered to, depending on how difficult it was to find suitable images.

Most importantly, we only chose candidate images with a public domain or CC0 copyright license. We further focused on images with the desired object concept as the central and dominant image component, however, the photograph could also include additional object concepts in the background. For instance, body parts were permitted in images of clothing parts, while human faces were generally avoided due to their strong salience (except for concepts like “man” and “woman,” which are defined by human faces). In addition, we took care to select pictures that still contained the majority of the object after cropping to a square size.

We selected images of objects with naturalistic backgrounds, i.e., we avoided images with uniform-colored backgrounds and excluded pictures in which the background was removed or recognizably modified. We avoided blurry images with over- or underexposed lighting and excluded pictures with non-naturalistic colors (including grayscale) or strong color filters. Finally, we avoided images with borders, watermarks, added text, or text that naturally appeared within the image, especially when the text referred to the concept’s name (e.g., “toothpaste” written on a toothpaste). Since for some concepts this was very difficult to avoid, we edited the color and exposure with photo editing software when necessary.

#### Identification of candidate images

Candidate images were manually selected from the photography websites *Flickr*, *Pexels*, *Pikrepo*, *Pixabay*, and *Wikimedia commons*. Search terms constituted the label of the object concept or synonyms. In some cases, foreign translations of the labels were used as keywords. Further, we added our own images for concepts for which no adequate image was available and uploaded them on Flickr with a CC0 license.

#### Image cropping and manual quality check

All candidate images were cropped to a square size using Adobe Photoshop. Next, the images were manually screened, and all images that were of low quality or did not meet the selection criteria were removed. The previous steps were repeated for all images until we found one suitable candidate image for every concept.

#### Semi-automatic identification of highly similar or duplicate images

We passed all pictures through the deep convolutional neural network VGG-16 (Simonyan & Zisserman, 2014) to ensure that candidate images were novel and not yet included in the THINGS database. All duplicate images were exchanged with new candidate images that underwent the entire selection process again. We manually checked all images once more and, if necessary, repeated the previous steps. Finally, all images were compressed to a maximum size of 1600×1600 pixels, converted to jpeg format for consistency with THINGS images, and named according to the respective THINGS’ unique concept ID. After cropping, all photographs had a minimum of 480×480 pixels, but on average the images were 1467.33 (*SD* = 266.99) pixels or larger.

### Identification of additional higher-level categories

THINGS includes membership information of 1854 object concepts for 27 common higher-level categories. In the present study, we extended the number of categories to 53 by identifying 26 additional superordinate categories. We employed the same dataset that was used to identify the initial 27 categories (see Hebart et al., 2019). In short, the original 27 high-level categories were identified according to the following steps: (1) Workers on AMT proposed higher-level categories for all 1854 object concepts (*n* = 20 per concept). (2) Another group of participants (*n* = 20 per concept) selected the most suitable category for each object from those candidate terms to reduce noise. (3) After correcting for spelling errors and unifying synonyms, superordinate categories were kept if 11 or more workers agreed on the high-level category or if five or more workers agreed on the high-level category while all others were named a maximum of two times. (4) All categories with a minimum of 15 members named consistently by workers were retained. This original procedure resulted in 27 higher-level categories (for more detail, see Hebart et al., 2019).

For THINGSplus, additional categories were identified by using a less restrictive criterion, using a minimum of 6 members rather than 15 members, since workers may not frequently name common categories even though these categories would accurately describe their members (e.g., for “deer”, workers may agree on “animal” but not “mammal”, while for “dog”, they may agree on “mammal” but not “animal”). Based on this lenient criterion, we identified 84 high-level categories. Of these categories, we merged five: “boat” was integrated into “watercraft”, “sea creature” into “sea animal”, “hair tool” into “hair accessory”, “game” into “entertainment”, and “craft supply” and “art supply” were merged into “arts & crafts supply”. Finally, we removed four additional categories that were already included in other categories and did not differ sufficiently: “storage” overlapped with “container”, “accessory” with “clothing accessory”, and “decoration” and “holiday decoration” with “home decor”. Finally, we removed “underwear” since it was considered too explicit. This left us with 74 high-level categories. Finally, rather than relying solely on agreement of AMT workers, memberships of all categories were independently assigned by two of the authors (L.M.S. and J.P.), and inconsistencies were corrected by the third author (M.N.H.). Of this expanded list, all superordinate categories with 15 members or more were kept, resulting in a total of 53 categories.

### Creation of concept-specific and image-specific norms and metadata

#### Participants

A total of 9263 individuals from AMT were recruited for different tasks, including object image labeling and rating of typicality, size, and several other semantic and perceptual properties of object concepts. All participants resided in the USA.

The experiment was approved by the Ethics Committee of the Medical Faculty of Leipzig University, and workers were compensated with small reimbursements for solving short tasks (labeled “Human Intelligence Task,” HIT) related to our study. Individual workers often participated in several HITs in a row, i.e., they potentially engaged in the same task multiple times. A limitation of unsupervised crowdsourcing is that some participants may not comply with task instructions. For this reason, we defined several criteria to identify workers who did not participate conscientiously in the experiment (see exclusion criteria below). In the following, we will refer to these participants as “non-adherent workers.” After exclusion, 8456 individual workers remained (4924 female, 3489 male, 53 other), who completed a total of 299,898 HITs (= 719,804 trials; 1 to 2345 HITs per worker, M = 35.46, SD = 73.60). The mean age of the sample was 37.24 years (*SD* = 12.06, 18 to 86 years). Demographic information for each task is summarized in Table 1**.** Please note that some workers participated in several experiments but are only counted once in the above statistics.

**Table 1:** Participant statistics pre- and after exclusion and number of single trials per experimental task

Task	Pre-exclusion		Post-exclusion				
	*N* Worker	*N* Trials	*N* Worker	*N* Trials	Gender	*M* Age	*SD* Age
Image labeling	1956	522,140	1956	467,906	female: 1283 male: 659 other: 14	37.72	12.03
Object size	2162	93,210	2010	82,990	female: 1165 male: 830 other: 15	37.47	12.22
Object typicality	1318	14,555	1201	13,024	female: 747 male: 443 other: 11	36.10	12.10
Object properties	4360	74,160	4156	69,182	female: 2418 male: 1712 other: 26	36.02	11.80
Arousal follow-up collection	1458	111,240	1040	86,702	female: 528 male: 510 other: 2	45.55	10.30

The sample size pre- and post-exclusion are identical for the Image Labeling task, as we excluded single trials instead of individual workers

##### Image labeling

For the image labeling task, participants who finished five trials faster than 800 ms or all ten trials faster than 1.10 s each were marked as candidates for non-adherent workers. Workers who wrote comments which have been related to low-quality responses in the past (e.g., “nice,” “good,” “thanks”) were also marked as candidates for being non-adherent. All participants labeled as candidates at least twice were prevented from further participation using an automated script created by a member of our team. After data collection, we did not exclude workers but individual HITs or trials (i.e., single labels) from our analyses matching the exclusion criteria reported in the analysis sections below.

##### Object typicality

Data for the typicality experiment were collected twice, as the initial version of the experiment did not control for the potential confound of familiarity (see *Design & Procedure* for more details). In the following, we will only report the results and design of the second, improved version of the experiment. However, information about both versions can be derived from Appendix 1. We also made the combined data available in the THINGS database.

Workers were only allowed to participate in 20 HITs. This was ensured by a JavaScript code generated through https://uniqueturker.myleott.com/. Participants who completed more than five trials below 30 s were excluded from data analysis. Mean typicality ranks were computed for every concept over participants, and the 5% of workers whose responses correlated lowest with the mean ratings across all their trials were excluded (cut off: *r*_*s*_ = .095).

##### Object size

As with typicality, for object size ratings, each individual worker could only participate 20 times. During data collection, workers who responded faster than 4.0 s at least five times or faster than 5.5 s in all ten trials were labeled non-adherent. Participants who wrote suspicious comments (see above) were also marked as non-adherent workers. Again, all participants identified as non-adherent candidates twice were prevented from participating in further HITs.

After data collection, additional HITs were excluded if we suspected that a subject answered randomly to finish the task quickly. This included HITs in which the worker did not know the object in at least four trials or mainly responded by clicking on a similar location of the rating scale, i.e., HITs with a response variance below 0.40 for the first step of the rating task. Further, size ratings were randomly shuffled over trials to generate a random distribution of ratings. For every HIT in the original and randomized data set we computed the deviation of every response to each object’s average size rating. All HITs that differed below the 20th percentile from the random variance distribution were excluded (i.e., HITs that were too similar to the random answer pattern, suggesting that the workers also answered randomly). Finally, we rejected all HITs of a given worker if half or more of that worker’s HITs were flagged as suspicious.

##### Object properties

Each worker was allowed to complete 40 surveys of the object properties task. Again, workers who wrote suspicious comments were marked as non-adherent, and all participants marked as non-adherent at least twice were prevented from participating in further HITs. After collecting the data, HITs completed faster than 1 s were removed. In addition, a HIT was excluded if the responses of the survey varied less than 0.50 between the 11 items or if the responses of all items differed 25 points or more from the median ratings of the currently sampled concept.

A closer look at the collected ratings showed that arousal level correlated negatively with pleasantness (*r*(1854) = *–* 0.77, 95% CI = *–* 0.75 to *–* 0.79). This suggests that participants interpreted the dimension of arousal mainly as negative arousal, as associated with fear or disgust evoking objects (e.g., weapons, spiders), and less with positively arousing concepts (e.g., puppies, gifts). For this reason, we collected arousal ratings again, with the intention of clarifying the concept of arousal further. All participants who wrote suspicious comments or either responded five trials faster than 1 s or ten trials faster than 1.5 s were marked as non-adherent workers. All workers labeled non-adherent at least twice were prevented from further participation. After data collection, we excluded all HITs in which the responses varied less than 0.25 and all HITs in which the ratings differed more than 30 units from the median answers.

#### Design & procedure

All image and object nouns were derived from the THINGS database. In general, participants were presented with a short instruction, after which they provided informed consent and agreed to the data storage policy before continuing with the HIT. At the end of each task, participants indicated their age and gender and were provided with the opportunity to leave comments before submitting the HIT.

##### Image labeling

We collected image labeling information for all 26,107 images of the THINGS image dataset. Each image was sampled at least 20 times. Participants completed HITs of ten trials. Each trial showed one image, and participants were asked to provide the name of the prominently depicted object as found in a dictionary. If they did not recognize the object, they were instructed to guess what it could be. If present, they were encouraged to name all additional objects in the image separately (e.g., objects depicted in the background). Participants responded by typing their answers in text fields below the image.

##### Object typicality

Overall, participants sorted 1448 of the 1854 object concepts based on how typical they are of the 53 high-level categories (as generated above). Together, all categories comprised 2355 members, since categories could overlap with each other (e.g., “animal” and “mammal”). Not all THINGS concepts were sampled, as some objects do not belong to any superordinate category. Each object concept was sampled at least 50 times. The THINGS database includes several concepts with identical labels but distinct meanings (homonyms; e.g., “bracelet” as an independent piece of jewelry vs. “bracelet” of a watch). Thus, we provided additional information for all homonyms in parentheses, e.g., “bracelet (jewelry)”, “bracelet (watch)”.

Participants were shown a higher-level category label (e.g., “animal”) at the top of the screen and eight randomly aligned objects nouns below that belonged to the category (e.g., “dog,” “parrot,” “zebra,” ...). Their task was to drag the concepts into a box, sorting them based on how typical or representative they were of the category. Typicality was explained by referring to the game show *Family Feud*: “More typical is what you think more people would say first, less typical is what fewer people would say.” Examples of three categories not included in THINGS were given to make the concept of typicality as clear as possible to participants.

In addition, participants could drag concept words into an unknown box instead of the typicality box to indicate that they were unfamiliar with the word’s meaning. The unknown box was introduced to reduce the confound of familiarity, i.e., unfamiliar words are rated as less typical because they are not known by some people (Malt & Smith, 1982).

##### Object size

Size ratings were collected for all 1854 object nouns. For words with ambiguous meaning (homonyms), additional information about the context of the concept was given in parentheses, e.g., “bat (sports)” versus “bat (animal).” Each object concept was sampled at least 50 times. The object concept “straw (stalks of dried grain)” was not sampled in the first round of data collection by mistake, while the concept “straw (for drinking)” was sampled twice as often. Thus, we collected 50 additional HITs, each including the object “straw (stalks of dried grain)” and each nine objects from the previous collection who had the smallest sample size after exclusion. After excluding low-quality trials, an average of 44.80 trials was included in analyses per concept (17 to 91 trials, *SD* = 2.62).

Each HIT comprised ten trials. Per trial, one object noun was shown, and participants were asked to rate the real-world size of the object in two steps. First, workers were instructed to indicate the approximate size of the object on a continuous scale (520 units). Nine object nouns were provided as response anchors for size references on the scale. As a reference, the objects “grain of sand,” “marble,” “chicken egg,” “grapefruit,” “microwave oven,” “washing machine,” “king-size bed,” “ambulance” and “aircraft carrier” were chosen, as they are relatively standardized in size and encompass the whole size range of all 1854 objects quite evenly assuming log scale. We did not give participants any further instructions on how to define size, i.e., through length, height, surface size, or volume. If participants did not know the object or if the object had no size (e.g., “sand,” “water”), workers could skip to the subsequent trial by checking respective response boxes below the scale.

In the second step, the rating scale zoomed closer, now encompassing 160 of the previous 520 units. For instance, if workers clicked on a position on the scale between “microwave” and “washing machine” in the previous step, the zoomed-in scale now ranged from one anchor point below (“grapefruit”) to one anchor point above the chosen interval (“king size bed”). Furthermore, one additional reference object was embedded between each of the previous anchor points (e.g., “football” between “grapefruit” and “microwave”; see. Appendix 2 for a list of all referenced objects). The participant’s task was to refine their initial response and indicate the size range that the object usually occupies in the real world. For example, some coconuts can be larger than others but are relatively similar in size. In contrast, boats can vary more widely, ranging from small rowboats to large fishing boats. Participants responded by clicking and dragging their mouse from the lower to upper end of the range. For objects with no size range (e.g., standardized objects like “soccer ball”), workers were asked to click on the scale only once. During step 2, workers always had the option to go back to step 1 to edit their previous choice.

##### Object properties

Participants completed short surveys, including the 1854 object nouns and 11 items related to properties of that object (“manmadeness,” “preciousness,” “liveliness,” “heaviness,” “naturalness,” “ability to move,” “graspability,” “holdability,” “ability to be moved,” “pleasantness,” and “arousal level”). Every object concept was sampled 40 times.

In each survey, participants were presented with one object noun and a corresponding image of the object concept. They were asked to rate how well the 11 different properties apply to the object using seven-point Likert scales. For properties “manmade,” “precious,” “something that lives,” “heavy,” “natural,” and “something that moves,” participants responded on a scale ranging from 1 “strongly disagree” to 7 “strongly agree.” The features “How difficult/easy is it to grasp?”, “How difficult/easy is it to hold?” and “How difficult/easy is it to move?” were rated on a scale from 1 “very difficult” to 7 “very easy.” Finally, workers responded to “How unpleasant/pleasant is the object?” on a scale ranging from 1 “very unpleasant” to 7 “very pleasant” and to “How calming or arousing/agitating is the object?” on a scale from 1 “very calming” to 7 “very arousing.”

We collected arousal again, due to their high correlation with pleasantness ratings. We used a similar task as described above, but this time, we explained the construct of arousal in more detail and emphasized its independence from valence. We did this by giving specific examples of very arousing or very calming objects with strong positive and negative valence. Two sets of example concepts were used throughout the data collection (pleasant: kittens & gifts vs. puppy & baby; unpleasant: spider & knife vs. snake & gun), whereby the concepts used as examples were not sampled in the respective version of the rating task. Each HIT comprised 11 trials. In each trial, participants were shown the image and name of one object and instructed to rate the object's arousal level on a scale from 1 “very calming” to 7 “very arousing.” Initially, every object concept was sampled 40 times. However, as we deemed the internal rating consistency insufficient (split-half reliability: *r* = 0.64), we collected another 20 samples per concept.

#### Analyses

All analyses were computed in Python 3 (van Rossum & Drake, 1995). In instances where the central tendency of multiple correlation coefficients was computed, we first standardized the coefficients using Fisher-z transform and then re-standardized the average.

##### Image labeling

First, trialwise answers were corrected in Python. All labels were changed into their singular form, provided that the THINGS concept noun was also written in the singular form (e.g., we corrected “dogs” to “dog” but not “jeans” to “jean”). Capital letters were changed to lower case letters (e.g., we corrected “DOG” or “Dog” to “dog”), and we removed nonsense answers (e.g., “sjbfk”), indefinite articles (e.g., “a dog” for “dog”), punctuation marks (e.g., “dog?”), additional text (e.g., “the image shows a dog”), and comments (e.g., “sorry, I do not know this object”).

Next, the data was manually corrected for spelling and typing errors (e.g., we corrected “ardvark” or “aardvsrk” to “aardvark”). Abbreviations (e.g., “tv” for “television”) or different ways of spelling (e.g., “yoyo,” “yo yo,” or “yo-yo”) were assumed to reflect the same concept and manually adapted to the spelling of the THINGS nouns. Finally, we created an additional version of the responses in which we adapted more specific but otherwise identical labels to the respective spelling of the THINGS concept (e.g., corrected “green acorn” to “acorn” or “electric air conditioner” to “air conditioner”).

We computed nameability and naming consistency measures for each image. Nameability was defined as the proportion of generated labels identical to the respective THINGS noun. Naming consistency refers to the proportion of the most used label, regardless of whether it was correct.

##### Object typicality

For every trial, each word was assigned a rank from 1 (most typical) to 8 (least typical), following the order participants has sorted them in. The maximum ranks varied in some trials, depending on how many words were classified as unknown. For this reason, ranks were divided by the maximum rank for that trial, resulting in standardized ranks from 0 (most typical) to 1 (least typical). This way, the lowest possible score (highest typicality) could vary between trials (e.g., 1/8 for trials with zero unfamiliar objects, 1/6 for trials with two unknown objects). Hence, the typicality scores are best interpreted at the ordinal level. To avoid misinterpretations the scores were inverted, i.e., 0.875 was defined as most typical and 0 as least typical. Finally, the average typicality score and standard deviations were computed for each concept.

Internal rating consistency of typicality ratings was assessed for every higher-level category separately. More precisely, we computed the split-half reliabilities by randomly dividing the trialwise data into two sets and correlating the mean size scores and mean size ranges of each member between the two halves (*n* = 2355). This procedure was repeated 30 times, and the resulting correlation coefficients were standardized using the Fisher-z transform before averaging over iterations. The mean correlations were then re-transformed and corrected for split-half reliability using Spearman–Brown correction.

##### Object size

For object size, we first computed trialwise object sizes. The size of each object was defined as the midpoint of the size range collected in the second step of the rating procedure. If no range was indicated in the respective trial, the point on the rating scale on which the respondent clicked was taken as the size measure. Next, we averaged the responses over trials and computed mean size rating, size range, and start and endpoint of the range for each object concept. Standard deviations were calculated for all four size measures. Split-half reliabilities were computed analogously to the typicality ratings.

##### Object properties

We calculated mean rating and standard deviations of all object properties (including new arousal ratings) for each object concept. Further, we determined the absolute rating frequencies (at each scale level) of all 1854 concepts and split-half reliabilities for each object property.

## Results

### Fifty-three higher-level categories

Of the 1854 object concepts, 1448 were assigned to one or more of the 53 higher-level categories. Conversely, 406 objects did not belong to any of the THINGS higher-level categories (e.g., “altar,” “backdrop,” “fire”). The overlap between categories was moderate. Overall, 664 concepts belonged to two or more higher-level categories. Of these, 476 were assigned to two different categories, 140 to three, 42 to four, five objects to five, and one object (“chainsaw”) to six distinct categories.

**Fig. 1:**
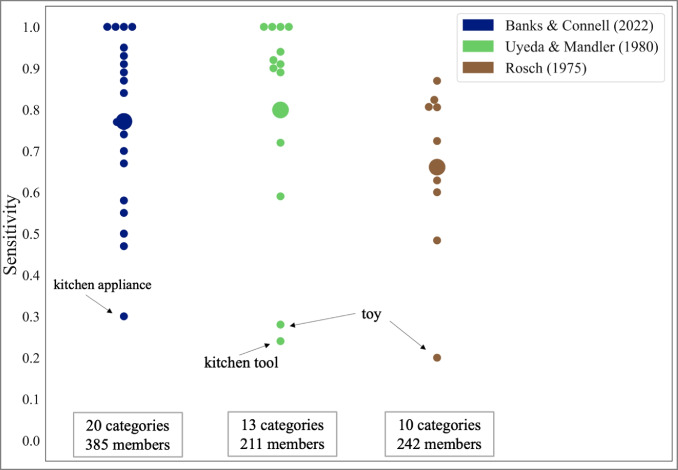
Sensitivity of the THINGSplus categorizations in comparison to three datasets. *Note*. Categorization sensitivities per higher-level category, defined as the fraction of all shared objects categorized equally in THINGSplus as in the external dataset. We only computed the sensitivity for concepts matching between both datasets

To validate the higher-level categories, we determined their congruence with categorizations in three other datasets (Banks & Connell, 2022; Rosch, 1975; Uyeda & Mandler, 1980). Some category names included in the external studies were slightly different from our category names but conveyed the same meaning (e.g., “beverage” instead of “drink,” “animals” instead of “animal”). In these cases we treated them as reflecting the same higher-level category. Not all concepts in the respective dataset overlapped with those included in THINGSplus, therefore we only considered matching objects in the comparison. We then computed classification sensitivity per higher-level category, defined as the fraction of category members included in the external dataset also identified as category members in our database. Table 2 summarizes the sensitivities per category compared to the three datasets (see also Fig. 1). Table 2 also provides examples of objects only assigned to a specific category in THINGSplus versus solely in the other dataset.

**Table 2:** Sensitivities of shared THINGSplus categories and number of uniquely categorized, equally categorized, and not-categorized object concepts in comparison to three datasets

Category	*N* unique THINGS	*N* overlap	*N* unique other	Sensitivity	Unique THINGS	Unique other
Animal	30	91	0	1.00	alpaca, blowfish, bull, calf, catfish, …	
Bird	2, **0**, *0*	20, **12**, *20*	0, **0**, *3*	0.95, **0.92**, *0.87*	bird, rooster	**chicken (meat)**, *crane, bat*
Body part	0, **0**	28, **18**	0, **2**	1.00, **0.90**		**nail, trunk**
Clothing	5, **0**, *1*	30, **20**, *25*	3, **0**, *6*	0.91, **1.00**, *0.81*	bonnet, bracelet, crown, sombrero, tiara, *bow*	bag, cap, glasses, *handkerchief, purse, ring, watch, necklace*, …
Drink	**0**	**7**	**0**	**1.00**		
Farm animal	1	14	5	0.74	calf	alpaca, cat, dog, fish, llama
Fruit	0, **0**, *0*	28, **26**, *28*	2, **0**, *6*	0.93, **1.00**, *0.82*		cucumber, squash, *pumpkin, nut, gourd, olive, pickle*, …
Furniture	1, **0**, *0*	11, **10**, *15*	9, **7**, *16*	0.55, **0.59**, *0.48*	chest	computer, cooker, door, fireplace, freezer, …, **lamp, buffet, piano, stereo, television,** …, *stove, clock, refrigerator, vase, ashtray, *…
Garden tool	0	12	6	0.67		brush, bucket, drill, fork, ladder, …
Headwear	3	4	4	0.50	hat, headband, tiara	baseball, bucket, cap, straw
Insect	1	14	6	0.70	bug	slug, snail, snake, spider, stick, …
Jewelry	0	10	3	0.77		diamond, gold, ruby
Kitchen appliance	1	8	19	0.30	cooker	bowl, dryer, fan, flan, fork, …
Kitchen tool	1, **0**	9, **5**	10, **16**	0.47, **0.24**	grinder	blender, blowtorch, bowl, fork, pan, …, **pot, mixer, plate, bowl, blender**, …
Musical instrument	0, **0**	24, **19**	0, **0**	1.00, **1.00**		
Personal hygiene item	0	7	5	0.58		cream, hair, highlighter, mirror, powder
Sea animal	2	16	2	0.89	fish, snail	goldfish, horse
Sports equipment	*5*	*4*	*2*	*0.67*	*ball, bat, skateboard, sled, surfboard*	*squash, checkers*
Tool	11, **7**, *4*	21, **15**, *22*	4, **1**, *15*	0.84, **0.94,** *0.63*	blowtorch, brush, fork, grater, hoe, …, **chain, fork, ladle, punch, rope,** …, *chain, pitchfork, razor, rope, spatula,*…	ball, nail, paper, stapler, wood, toolbox, bench, **wood,** *lumber, brace, …*
Toy	**1**, *3*	**5,** *6*	**13**, *24*	**0.28**, *0.20*	**scooter,** *scooter, skateboard, slingshot*	**block, wagon, truck, tricycle, train, **…, *baseball, drum, football, game, swing, *…
Vegetable	3, **0**, *1*	20, **17**, *21*	3, **2**, *8*	0.87, **0.89**, *0.72*	jalapeno, parsley, squash, *gourd*	avocado, mushroom, rocket, **bean, rice,** *bean, mushroom, avocado, sauerkraut, seaweed,* …
Vehicle	1, **0**, *0*	19, **21**, *29*	0, **2**, *7*	1.00, **0.95**, *0.85*	rocket	**trailer, tank,** *trailer, tank, horse, blimp, camel,* …
Weapon	3, **2**, *2*	19, **13**, *21*	6, **5**, *14*	0.76, **0.72**, *0.60*	blowtorch, chainsaw, scissors, **axe, bat,** *bat, crowbar*	bottle, brick, glass, rock, rope, …, **chain, stick, rock, rope, hand,** *hatchet, razor, rocket, stick, rock,* …

Results for the comparison of THINGSplus and Banks and Connell (2022) are printed in regular font, in **bold** for the comparison to Uyeda and Mandler (1980), and in *cursive* for Rosch (1975)

Additionally, we determined the sensitivity for all possible combinations of “test” and “true” datasets (see Table 3). Overall, categorization sensitivities were higher for the external datasets. However, as shown in Table 2, this is due to the more liberal categorizations in the external datasets. For instance, assigning “snail” to the category “insect” or “truck” to “toys.”

**Table 3:** Mean sensitivity per dataset in comparison to all other datasets

Truth	THINGSplus	Rosch (1975)	Banks and Connell (2022)	Uyeda and Mandler (1980)
Test				
THINGSplus	1.00	0.68	0.82	0.74
Rosch (1975)	0.88	1.00	0.95	0.99
Banks and Connell (2022)	0.90	0.87	1.00	0.95
Uyeda and Mandler (1980)	0.95	0.93	0.81	1.00

We only analyzed higher-level categories included in THINGSplus. Of these categories, Rosch (1975) have eight categories and 226 members in common with Banks and Connell (2022) and nine categories and 238 members with Uyeda and Mandler (1980). Banks and Connell (2022) and Uyeda and Mandler (1980) shared 11 categories and 224 members

### Image labeling

Table 4 summarizes the descriptive statistics of the Image Labeling experiment. Labeling data was not externally validated, as the data referred to individual images for which there was no adequate measure of comparison.

**Table 4:** Image- and object-wise percentiles and means of the naming consistency and nameability scores and number of concepts with one, five or ten highly accurate images

**Image-wise:**					Fraction of images with accuracy:		
	M	SD	Mean SE	Max. SE	≤ 10%	≥ 50%	≥ 90%
Naming consistency	0.70	0.23	0.09	0.27	0.00	0.53	0.25
Nameability	0.66	0.29	0.08	0.14	0.04	0.46	0.25
**Object-wise:**					Fraction of concepts with accuracy:		
	M	SD	Mean SE	Max. SE	≤ 10%	≥ 50%	≥ 90%
Naming consistency	0.70	0.25	0.03	0.03	0.00	0.60	0.20
Nameability	0.65	0.20	0.02	0.03	0.02	0.51	0.20
	Number of objects that have at least 1, 5, or 10 images with accuracy:						
	≥ 50%			≥ 80%			
	1	5	10	1	5	10	
Naming consistency	1800	1558	1214	1301	874	557	
Nameability	1679	1427	1119	1248	876	561	

Across all individual images, concepts were correctly labeled (nameability) 66% of the time, and nearly half of the images were correctly identified by at least 50% of AMT workers. Participants generally agreed on the name of the depicted object, whether correct or not, in 73% of cases (naming consistency).

Computing mean nameability per concept (averaged over image examples) showed that concepts were correctly named by 70% and consistently named by 71% of participants.

It is worth noting that nameability and naming consistency are rather conservative measures, and the present results are not surprising, given the large number of possible synonyms people can use instead of the THINGS nouns. Nevertheless, the concepts had 9.73 images on average with a reasonable nameability of ≥ 50% (*SD* = 5.10), or 10.55 images with a naming consistency of 50% or higher (*SD* = 5.70).

### Object typicality

Internal consistency of typicality ratings was determined separately for every higher-level category. Inspected over all 53 categories, consistency scores revealed high reliability averaged over all categories, M = 0.97 (SD = 0.01, *r*(2355) = 0.92*–*0.99; see Table 5).

**Table 5:** Overview of the categories, their most and least typical members and ratings consistency

Category	*N* member	Most typical member	Least typical member	Rating consistency (Pearson *r*)
animal	177	dog (0.85)	coral (0.02)	0.98
arts and crafts supply	44	paint (0.78)	marble (0.14)	0.96
bird	27	eagle (0.70)	puffin (0.11)	0.95
body part	34	arm (0.80)	beard (0.04)	0.97
breakfast food	35	egg (0.81)	French fries (0.06)	0.97
candy	16	candy (0.73)	marshmallow (0.15)	0.96
clothing	108	jeans (0.84)	straitjacket (0.05)	0.98
clothing accessory	38	belt (0.78)	eye patch (0.08)	0.97
condiment	15	ketchup (0.85)	applesauce (0.11)	0.98
construction equipment	28	bulldozer (0.71)	pump (0.15)	0.96
container	105	box (0.83)	tent (0.10)	0.97
dessert	37	cake (0.81)	cheese (0.02)	0.97
drink	19	coffee (0.75)	eggnog (0.05)	0.96
electronic device	74	laptop (0.84)	slicer (0.10)	0.97
farm animal	18	cow (0.81)	bison (0.10)	0.98
fastener	32	zipper (0.61)	gasket (0.09)	0.92
food	295	cheeseburger (0.84)	poppy (0.04)	0.97
footwear	15	shoe (0.86)	flipper (0.07)	0.99
fruit	34	apple (0.84)	mulberry (0.07)	0.98
furniture	39	couch (0.80)	lectern (0.08)	0.98
game	19	board game (0.71)	yo-yo (0.12)	0.96
garden tool	17	rake (0.71)	pickax (0.15)	0.98
hardware	79	bolt (0.72)	strainer (0.15)	0.92
headwear	19	hat (0.84)	headdress (0.20)	0.96
home appliance	38	stove1 (0.78)	soda fountain (0.10)	0.98
home decor	45	lamp (0.8)	abacus (0.07)	0.98
insect	17	fly (0.74)	earwig (0.10)	0.97
jewelry	15	necklace (0.77)	barrette (0.17)	0.98
kitchen appliance	20	refrigerator (0.77)	slicer (0.14)	0.98
kitchen tool	27	knife (0.79)	icepick (0.08)	0.98
lighting	16	lamp (0.81)	penlight (0.18)	0.98
mammal	88	dog (0.84)	aardvark (0.05)	0.97
medical equipment	27	stethoscope (0.77)	eye patch (0.17)	0.96
musical instrument	33	guitar (0.84)	chime (0.11)	0.98
office supply	25	pen (0.79)	punch2 (0.12)	0.96
outerwear	16	jacket (0.83)	lab coat (0.14)	0.98
part of car	30	steering wheel (0.77)	roof rack (0.09)	0.97
personal hygiene item	31	soap (0.81)	flatiron (0.15)	0.98
plant	47	flower (0.77)	leek (0.14)	0.96
protective clothing	16	helmet (0.69)	overalls (0.22)	0.95
safety equipment	21	helmet (0.70)	spacesuit (0.08)	0.95
school supply	26	notebook (0.78)	inkwell (0.01)	0.97
scientific equipment	35	microscope (0.81)	prism (0.16)	0.96
sea animal	30	dolphin (0.78)	barnacle (0.08)	0.97
seafood	24	fish (0.81)	sea urchin (0.09)	0.98
sports equipment	64	ball (0.80)	baton3 (0.05)	0.97
tool	107	hammer (0.84)	quill (0.09)	0.96
toy	34	ball (0.78)	stilt (0.06)	0.97
vegetable	42	carrot (0.78)	rhubarb (0.08)	0.97
vehicle	70	car (0.86)	rocket (0.14)	0.97
watercraft	19	boat (0.79)	torpedo (0.06)	0.97
weapon	48	gun (0.85)	trident (0.12)	0.97
women's clothing	20	dress (0.82)	boa (0.05)	0.99

We corrected the split-half correlations using the Spearman–Brown formula

For external validation typicality scores were Spearman rank correlated with equivalent norms of three different datasets:

1. Typicality ratings of 27 superordinate categories were compared to ratings collected by Hebart et al. (2020). In their study AMT workers were instructed to rate the typicality of 1619 THINGS objects using a Likert scale from 0 (atypical) to 10 (typical). The comparison revealed a high correlation of both norms’ typicality ratings, *r*_*s*_(1619) = 0.88 (95% CI = 0.87*–*0.89).
2. Next, typicality scores were compared to a dataset by Rosch (1975). Their dataset includes 207 objects categorized in accordance with THINGS into ten categories (see also Table 2). Typicality scores of objects for their respective category were rated on a Likert scale from 1 (typical) to 7 (atypical). We inverted the scores of Rosch (1975) for easier comparison. Typicality ranks of the two datasets were moderately to highly correlated, *r*_*s*_(207) = 0.74 (95% CI = 0.67*–*0.80).
3. Finally, we compared our data to typicality scores by Uyeda and Mandler (Uyeda & Mandler, 1980; see also Table 2). Their study comprises typicality ratings from 1 (typical) to 7 (atypical) for 13 categories and 199 concepts that match the THINGS categorization. Their inverted typicality scores showed a moderate correspondence with our data, *r*_*s*_(199) = 0.72 (95% CI = 0.64*–*0.78).

### Object size

Across all object concepts, mean size ratings varied from 100.02 to 423.10 on a scale from 0 to 519 (M = 235.80, SD = 57.73). Size ranges spanned between 4.82 to 78.60 units (M = 22.87, SD = 8.34). Size ratings showed a high internal consistency of Pearson *r*(1854) = 0.99 (95% CI = 0.992–0.993). The consistency of size range scores was also high, *r*(1854) = 0.87(95% *CI* = 0.85–0.88).

For external validation, THINGS size data was compared to size ratings collected by Konkle and Oliva (2011). In their study, perceived object size was determined by asking participants to sort 100 images of objects into eight groups with ascending real-world sizes. In addition, their dataset provides information about the actual size of objects. Actual object size was defined as the logarithmized diagonal of the object bounding boxes, as quantified by their length, height, and width in centimeters. We correlated our size ranks with Konkle & Oliva’s subjective size ranks for all concepts included in both datasets. The results showed a high correlation of Spearman *r*_*s*_(73) = 0.95 (95% CI = 0.92–0.97). Compared to their actual size measure, the correlation was high and significant as well, Pearson *r*(73) = 0.97 (95% CI = 0.95–0.98).

### Object properties

**Fig. 2:**
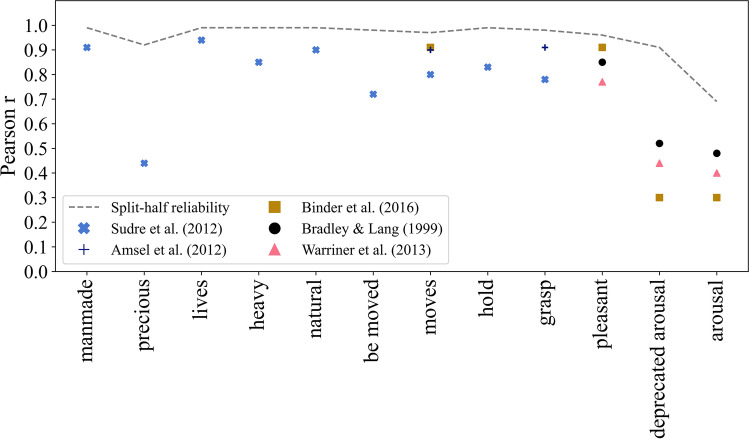
Correlation of object properties with external datasets and internal rating consistencies

The internal consistency of object property ratings was computed for each property individually, following the same procedure for typicality and size ratings. All properties collected in the initial questionnaire study showed a high split-half reliability of M = 0.98 (SD = 0.03, *r* = 0.91*–*0.99). In contrast, the consistency of subsequently collected arousal ratings was only moderate, even after increasing the number of samples per object by 50% (*r* = 0.69, 95% CI = 0.67–0.72). Object properties were externally validated using five different databases (Amsel et al., 2012; Binder et al., 2016; Bradley & Lang, 1999; Sudre et al., 2012; Warriner et al., 2013). Results of the external validations are summarized in Fig. 2 (see Appendix 3 for exact numbers). Across all 11 items, correlations were medium to high, again with the worst fit for arousal ratings.

The new arousal ratings were still moderately correlated with pleasantness (*r*(1854) = - 0.51). Nevertheless, this bias was significantly smaller than for the deprecated arousal ratings (*r*(1854) = - 0.77), demonstrating that the construct of arousal was better conveyed to the workers. Both arousal ratings were moderately correlated, *r*(1854) = 0.59.

## Discussion

With THINGS (Hebart et al., 2019) we have previously provided a large-scale database of 1854 concrete and picturable object concepts, 26,107 images of those concepts, and membership information for 27 higher-level categories. In the present work, we present THINGSplus, a large-scale extension to the THINGS database. We identified 26 additional higher-level categories and collected typicality ratings for members of all 53 categories. Further, we used crowdsourcing to generate a broad set of object property ratings; including manmadeness, preciousness, animacy heaviness, naturalness, ability to move or be moved, graspability, holdability, pleasantness, and arousal level. Moreover, we collected ratings of objects’ perceived size and size range. In addition, we asked people to name the most prominent as well as all other objects depicted in the 26,107 images to compute measures of image nameability and naming consistency. Finally, we collected one novel public domain image per concept. Our norms and metadata show high rating consistencies, demonstrating that object and image properties were reliably rated over participants. Moreover, we compared our collected norms to datasets created by other authors, confirming their external validity.

### Possible applications of THINGS and the newly collected norms, metadata, and public domain images

THINGS has already become a widely used resource by researchers. For example, the THINGS initiative (https://things-initiative.org/) brings together laboratories from different disciplines which share the same goal of understanding object recognition, semantic memory, and the content of mental object representations. Using the same database is advantageous, as it facilitates comparison between studies. It also allows sharing of data with other researchers. For example, the THINGS database is accompanied by extensive sets of freely available neuroimaging and EEG data (e.g., Gifford et al., 2022; Grootswagers et al., 2022; Hebart et al., 2023), behavioral similarity judgments (Hebart et al., 2020), memorability scores (Kramer et al., 2022) and feature production norms of the objects generated with the natural language model GPT-3 (Hansen & Hebart, 2022).

The newly collected norms and metadata represent an important expansion of the THINGS database. Normed data is crucial for characterizing objects and provides researchers with a standardized and detailed approach for selecting suitable stimuli. For instance, studies interested in emotional object processing or selective attention might focus on object concepts with high arousal levels and strong positive or negative valence (Bradley & Lang, 1994; Lang et al., 1997). Other studies might contrast neural responses to objects of different sizes or animacy (Chao et al., 1999; Konkle & Caramazza, 2013; Magri et al., 2020) with the prospect of identifying organizational dimensions of object representations and their related brain regions.

Image-specific nameability and naming consistency measures provide information on how well each image captures the desired object concepts and supports avoidance of object concepts that are ambiguously identified. We also collected labels for all other objects in the images, which facilitate identifying, for example, all images depicting human body parts or other confounding concepts (Downing et al., 2001; Downing et al., 2004).

Normative data also helps quantify variables that might exert confounding effects. For example, object naming studies have shown better performance in naming non-living objects than animals (Humphreys et al., 1988; Warrington & Shallice, 1984). However, when controlling for manipulability – in the present work defined by graspability and holdability *–* the benefit was larger for living and manipulable objects (Filliter et al., 2005).

Moreover, information about higher-level category memberships is crucial, as categorization is a vital ability of the cognitive system that allows us to make sense of the world around us. Our comprehensive set of 53 categories and associated membership information enables us to study processes related to category classification and to cluster conceptually similar concepts (e.g., compare animals to tools). Further, category affiliations are helpful for a wide range of experimental tasks, including object categorizations or verifying category production tasks.

Finally, when working with the THINGS database, our newly curated license-free images can be freely edited, can be used as example images in publications, and thus serve also as an important tool for data visualization and explorative analyses.

### Limitations of THINGSplus

Despite the high value and applicability of our newly collected norms, images, and related metadata, the THINGSplus project has some limitations.

First, different THINGSplus norms were acquired and preprocessed by different authors, and as a consequence, exclusion criteria for low-quality HITs varied. Nevertheless, cut-offs were determined quite rigorously and systematically, in most cases based on the distribution of deviations from the expected value, and even if the same criteria had been used, it is not clear how differences in tasks would translate to required changes in cut-offs. In the future, it would be possible to systematically evaluate the effect of exclusion criteria on the internal consistency and external validity of the collected norms.

Second, while typicality scores and most feature ratings showed high external validity, arousal ratings correlated only moderately with other arousal measures. We also observed a bias toward higher arousal levels for unpleasant (e.g., weapons, snakes) compared to pleasant (e.g., kittens, gifts) objects. This negativity bias persisted after collecting the ratings again with improved instructions.

Further, images without copyright restrictions were challenging to identify for some concepts, and in some cases, it was also infeasible to create our own photographs. For this reason, we sometimes had to choose images with slightly lower quality (e.g., with the object slightly blurred or not fully depicted after cropping).

Finally, image labeling data was manually corrected for spelling errors, alternative spelling, and abbreviations, introducing a certain degree of subjectivity. As a result, the nameability scores might underestimate the true nameability of objects.

### Open questions and future directions

We aim to further develop and improve the THINGS database. While we already collected a measure of nameability, including a more general measure of concept identifiability would allow for spotting hard-to-identify images or generally ambiguous concepts. With identifiability, we aim to capture the extent to which people grasp the meaning of a concept, even if they lack the appropriate label. Lexical databases like WordNet (Fellbaum, 1998) comprise only a limited selection of synonyms and categorical relations. Thus, for the future, it would be valuable to evaluate all generated image labels manually, to determine the portion of correct (e.g., “appetizer”), synonymous (e.g., “hors d’oevre,” “starter”), or more precise labels (e.g., “canapé”), as well other cases in which participant may have recognized the concept but did not know the correct name (e.g., “pre-meal snack”). We are currently in the process of developing this measure with the aim of adding it to THINGSplus.

Further, object concepts can be characterized by multiple features not yet included in this THINGSplus project. Some researchers might benefit from a measure of occurrence frequency, which provides insight into objects’ subjective relevance in everyday life and how representations of more frequently perceived concepts (e.g., “cow,” “pants”) differ from rare or less essential objects (e.g., “sea urchin,” “suspenders”). Further, we intend to acquire image-specific parameters, including curviness and rectilinearity ratings, potentially with other low-level features such as image clutter and degree of structure vs. object likeness. Moreover, knowing both the location and size that objects occupy in an image is crucial for many vision experiments. To this end, a typical approach is to ask participants to segment images in a way that separates the objects from their background. This is usually accomplished by dividing the pixels of an image into parts that have similar features and attributes or that depict specific objects. Asking human participants to segment THINGS images would provide the location of objects and their boundaries and thus increase manipulatory control.

While the distribution of workers from Amazon Mechanical Turk nicely captures many demographics of the general population (Berinsky et al., 2012; Casler et al., 2013), workers are generally more highly educated. Further, all workers resided in the USA. Thus, it would be interesting to know how well these results would translate to other, non-US cultures. Finally, future studies can strengthen the validity of the THINGS database by providing neural and behavioral correlates of THINGS norms and metadata.

## Conclusions

Together, we believe THINGSplus to be an important addition to the THINGS database for the study of object concepts, object images, related norms and metadata. Many laboratories around the globe already use these concepts and images. With our newly collected norms, public domain images, and metadata, we hope to strongly improve the database's usefulness and thus increase its value for the research community. The more widely THINGS is applied, the easier it is to compare different studies, research groups, and disciplines. We believe that these combined efforts will allow us to more effectively tackle the questions of how we make sense of the world around us, recognize objects and interact with them in a meaningful way.

## Figures and Tables

**Fig. 3:**
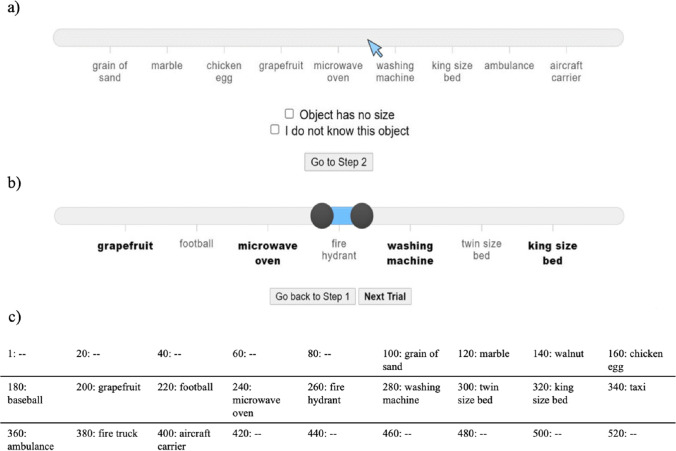
Layout of the size rating task. (**a**) In Step 1 of the rating task, participants were asked to rate the approximate size of an object in relation to nine reference objects. (**b**) In Step 2, the scale zoomed closer, now encompassing additional reference objects (light gray). Participants were asked to refine their answer and indicate the size range of the object. (**c**) List of all anchor points/reference objects and corresponding size score

## Data Availability

All norms, metadata, and supplementary public domain images of the THINGSplus project have been added to the existing THINGS database, which is freely available for academic purposes (https://osf.io/jum2f/).

## Appendix 1

### Combined data of both typicality task versions

#### Methods

Workers were only allowed to participate in 20 HITs of each version. Datasets from both versions of the typicality experiment were combined and analyzed jointly. Participants who completed more than five trials below 30 s were excluded from data analysis. Demographic information for both typicality versions is summarized in Appendix Table 6.

Each object concept was sampled at least 50 times per experimental version (100 in total). The design was identical to version 2, except for two aspects. First, we did not include an “unknown” box in the first version of the task. Second, we did not indicate additional information about homonyms in parentheses.

We did not control for homonymous members of the same category in the first version of the typicality task. Thus, we excluded all homonymous members of the same category in version1 and adapted the sorting ranks of the remaining members of the affected HIT accordingly (243 occurrences of ten concepts). The concept “gorilla” was mistakenly included as a member of the “food” category in the first experimental version and removed analogously to the homonyms (35 occurrences).

Mean typicality ranks were computed for every concept over participants, and the 5% of workers whose responses correlated lowest with the mean ratings across all their trials were excluded (cut off: *r*_*s*_= .024). The results are summarized in Appendix Table 7.

**Table 6:** Participant statistics pre- and post-exclusion for the combined typicality data

Task	Pre-exclusion		Post-exclusion				
	*N* Worker	*N* Trials	*N* Worker	*N* Trials	Gender	*M* Age	*SD* Age
Typicality Version 1 & 2	2466	29,088	2250	25,553	female: 1333 male: 902 other: 15	38.16	51.53

**Table 7:** Internal and external validation of the combined typicality data

Split-half reliability (Spearman–Brown corrected)	Mean *r* = 0.98	*SD* = 0.01	*r*(2355) = 0.95*–*0.99
External validation			
Hebart et al. (2020)	*r*_*s*_(1619) = 0.88	95% CI = 0.87–0.89	
Rosch (1975)	*r*_*s*_(207) = 0.74	95% CI = 0.67–0.79	
Uyeda and Mandler (1980)	*r*_*s*_(199) = 0.72	95% CI = 0.64–0.78	

## Appendix 2

Figure 3

## Appendix 3

### Split-half reliabilities of the object properties and correlations with other object norm databases

**Table:**

	Split-half reliability	Sudre et al. (2012)	Binder et al. (2016)	Bradley and Lang (1999)	Amsel et al. (2012)	Warriner et al. (2013)
N objects (*n* shared)		1000 (596)	535 (160)	1034 (178)	559 (411)	13,915 (1270)
Object property						
“manmade”	0.99	0.91	---	---	---	---
“precious”	0.92	0.44 (valuable)	---	---	---	---
“something that lives”	0.99	0.94	---	---	---	---
“heavy”	0.99	0.85	---	---	---	---
“natural”	0.99	-0.90 (manmade)	---	---	---	---
“How difficult/easy is it to move?”	0.98	0.72	---	---	---	---
“something that moves”	0.97	0.80 (is it fast)	0.91 (showing movement)	---	0.90	---
“How difficult/easy is it to hold?”	0.99	0.83 (can you hold it) 0.84 (hold in one hand)	---	---	---	---
“How difficult/easy is it to grasp?”	0.98	0.78 (can you hold it) 0.77 (hold in one hand)	---	---	0.91	---
“How unpleasant/pleasant is the object?”	0.96	---	0.91	0.85	---	0.77
“How calming or arousing/agitating is the object?”		---			---	
deprecated: new:	0.91 0.69		0.30 0.30	0.52 0.48		0.44 0.40

We corrected the split-half correlations using the Spearman–Brown formula

## Code availability

Code used for preprocessing and data analysis is available upon request.
